# Loss of USP28-mediated BRAF degradation drives resistance to RAF cancer therapies

**DOI:** 10.1084/jem.20171960

**Published:** 2018-07-02

**Authors:** Azad Saei, Marta Palafox, Touati Benoukraf, Nishi Kumari, Patrick William Jaynes, Prasanna Vasudevan Iyengar, Eva Muñoz-Couselo, Paolo Nuciforo, Javier Cortés, Christopher Nötzel, Nesaretnam Barr Kumarakulasinghe, John Lalith Charles Richard, Zul Fazreen Bin Adam Isa, Brendan Pang, Marta Guzman, Zhou Siqin, Henry Yang, Wai Leong Tam, Violeta Serra, Pieter Johan Adam Eichhorn

**Affiliations:** 1 Genome Institute of Singapore, A*STAR, Singapore, Singapore; 2 Department of Biochemistry, Yong Loo Lin School of Medicine, National University of Singapore, Singapore; 3 Vall d’Hebron Institute of Oncology (VHIO), Barcelona, Spain; 4 Cancer Science Institute of Singapore, National University of Singapore, Singapore; 5 Cancer Ramon y Cajal University Hospital, Madrid, Spain; 6 IOB Institute of Oncology, Quironsalud group, Madrid & Barcelona, Spain; 7 Department of Hematology-Oncology, National University Cancer Institute, Singapore, National University Health System, Singapore, Singapore; 8 Department of Pharmacology, Yong Loo Lin School of Medicine, National University of Singapore, Singapore; 9 School of Pharmacy and Biomedical Sciences, Curtin University, Perth, Australia

## Abstract

RAF kinase inhibitors are clinically active in patients with BRAF (V600E) mutant melanoma. However, rarely do tumors regress completely, with the majority of responses being short-lived. This is partially mediated through the loss of negative feedback loops after MAPK inhibition and reactivation of upstream signaling. Here, we demonstrate that the deubiquitinating enzyme USP28 functions through a feedback loop to destabilize RAF family members. Loss of USP28 stabilizes BRAF enhancing downstream MAPK activation and promotes resistance to RAF inhibitor therapy in culture and in vivo models. Importantly, we demonstrate that USP28 is deleted in a proportion of melanoma patients and may act as a biomarker for response to BRAF inhibitor therapy in patients. Furthermore, we identify Rigosertib as a possible therapeutic strategy for USP28-depleted tumors. Our results show that loss of USP28 enhances MAPK activity through the stabilization of RAF family members and is a key factor in BRAF inhibitor resistance.

## Abstract (precis)

Adaptive responses have been demonstrated to limit activity to targeted therapies. Saei et al. show that loss of USP28/FBW7-mediated BRAF degradation is observed in a proportion of melanoma patients and can be responsible for resistance through upregulation of MAPK signaling pathway.

## Graphical Abstract

**Figure:**
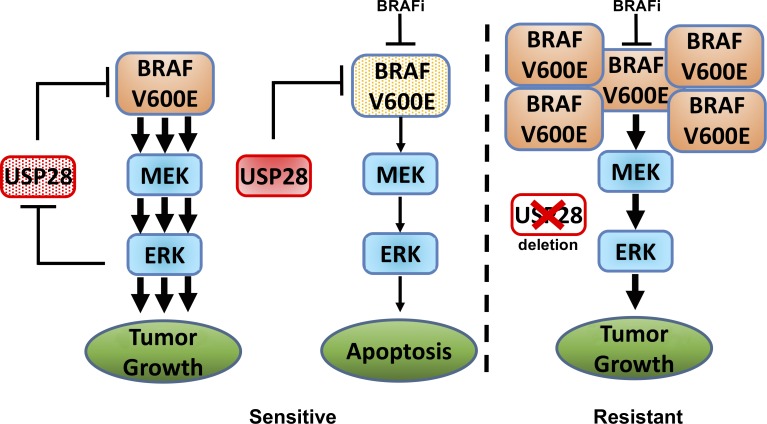

## Introduction

Activating mutations in the BRAF oncogene are found in 40–60% of patients with advanced melanoma (Davies et al., 2002). Targeted therapy of melanoma patients harboring BRAF (V600E) mutations with RAF and MEK inhibitors has markedly improved the outcome of this disease (Chapman et al., 2011; Larkin et al., 2014). Despite the survival gains provided by these therapies, most responses remain transient as a result of primary or acquired resistance. Interestingly, the majority of molecular lesions that prime resistance to MAPK inhibition result in the constitutive activation of downstream ERK signaling (Lito et al., 2013). These include up-regulation of receptor tyrosine kinases or growth factors (EGFR and ERBB3), activating mutations in MEK or NRAS, loss of expression of the NRAS negative regulator NF1, or the expression of alternatively spliced variants of BRAF (Nazarian et al., 2010; Poulikakos et al., 2011; Shi et al., 2012; Wilson et al., 2012; Abel et al., 2013; Whittaker et al., 2013; Sun et al., 2014). Importantly, MAPK pathway inhibitor resistance can also result from amplification and increased expression of BRAF or CRAF likely resulting in RAF dimerization with itself or its family members (Corcoran et al., 2010; Shi et al., 2012). Moreover, several functional genomic and next-generation sequencing–based approaches probing resistant models have identified COT/TPL2, STAG family members, loss of RNF125, and YAP overexpression as mechanisms of BRAF inhibitor resistance (Johannessen et al., 2010; Kim et al., 2015; Lin et al., 2015; Shen et al., 2016). However, these mechanisms are not prevalent enough to justify the high frequency of primary and acquired resistance to BRAF inhibitors.

Ubiquitin modification of MAPK signaling components is emerging as an important regulatory mechanism of MAPK pathway control (Laine and Ronai, 2005). It is well described that monoubiquitination and/or polyubiquitination resulting from the various assortment of ubiquitin chain topologies convey distinct structural and functional information to the targeted protein. For the most part, K48-linked chains serve to act as the prototypical degradation signal shunting the protein for proteasome mediated degradation, whereas K63-linked chains perform several nonproteolytic functions, including cellular signaling, DNA damage repair, intracellular trafficking, and ribosomal biogenesis (Komander and Rape, 2012). The conjugating function of E3 ligases is opposed by deubiquitinating enzymes (DUBs). There are ∼80 DUBs in the human proteome, and several these have been implicated in human pathologies, including cancer (Nijman et al., 2005). Nevertheless, the role of DUBs in MAPK pathway regulation remains ill defined (Kumari et al., 2017).

A common characteristic of both normal and transformed cell lines is the activation of both positive and negative feedback loops to continuously fine tune desired pathway activation and corresponding cellular responses (Lito et al., 2013; Rozengurt et al., 2014). For example, this may be achieved either through the up-regulation of receptor tyrosine kinases (EGFR and ERBB3) to maintain hyperactivation of the pathway or through the activation of inhibitory phosphatases (DUSP) to down-regulate the pathway (Pratilas et al., 2009; Chandarlapaty et al., 2011; Serra et al., 2011; Abel et al., 2013; Sun et al., 2014). Similarly, we reasoned that down-regulation of the MAPK pathway by targeted inhibition would alter the expression of certain DUBs, which would act through feedback loops to then retarget components of the RAS–RAF–MEK–ERK pathway. Here, we identify the DUB USP28 as a key regulator of MAPK activity. Biochemically, USP28 expression is enhanced after treatment with the BRAF inhibitor vemurafenib whereby USP28 acts in conjunction with FBW7 to regulate the stability of RAF family members. FBW7 is a component of SCF (complex of SKP1, CUL1, and F-box protein) ubiquitin ligase complex where FBW7 acts as a substrate recognition subunit mediating the turnover of multiple oncogenes involved in a wide range of human cancers (Welcker and Clurman, 2008). Under normal physiological conditions, FBW7 is autocatalytically ubiquitinated by the SCF complex resulting in its degradation. USP28 deubiquitinates and stabilizes FBW7 resulting in enhanced degradation of FBW7 substrates (Schülein-Völk et al., 2014). Recently, inactivating mutations in FBW7 have been identified in melanoma, correlating with poor prognosis (Aydin et al., 2014). Importantly, we demonstrate that USP28 expression is deleted in ∼10% of all melanoma patients, of which half of these patient’s harbor mutations in BRAF (V600E), NF1, or NRAS, supporting a role for USP28 loss in melanoma progression. Furthermore, depletion of USP28 promotes resistance to vemurafenib in vitro and in vivo and low USP28 expression is associated with a shorter time to progression in patients receiving combined BRAF/MEK inhibitor therapies. In addition, USP28-depleted cells are synthetic lethal with the RAF/PLK1 inhibitor rigosertib, suggesting rigosertib as a potential therapeutic strategy in USP28-depleted melanoma.

## Results

### Identification of USP28 as a negative regulator of MAPK signaling

**Figure 1.**
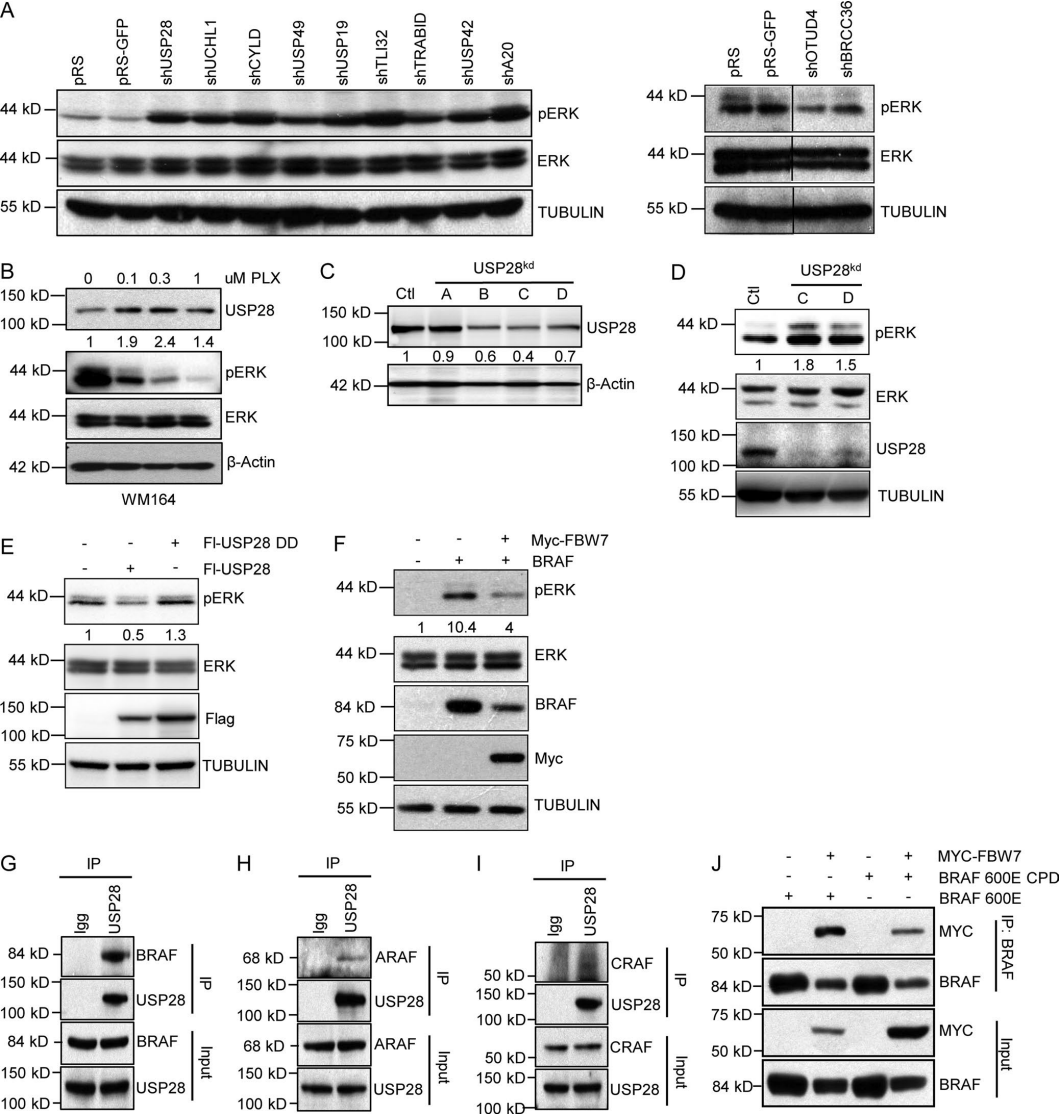
**Identification of USP28 as negative regulator of ERK signaling. (A)** Third round selection of DUB screen. Immunoblot analysis of 293T cells expressing shRNA vectors targeting the indicated DUBs. (**B)** Immunoblot analysis of WM164 melanoma cells treated with indicated concentrations of PLX4032 and probed with the indicated antibodies. **(C)** Immunoblot analysis of 293T cells expressing shRNA vectors (A–D) targeting USP28 and probed with the indicated antibodies. **(D)** Immunoblot analysis of 293T cells expressing USP28 shRNA vectors (C and D). Whole cell extracts were probed with the indicated antibodies. **(E)** Immunoblot analysis in 293T cells expressing Flag-USP28 or Flag-USP28DD. Whole cell extracts were probed with the indicated antibodies. **(F)** Immunoblot analysis showing 293T cells expressing Myc-FBW7 and BRAF. Whole cell extracts were probed with the indicated antibodies. **(G–I)** Immunoprecipitation of endogenous USP28 in 293T cells and an immunoblot analysis of indicated proteins BRAF (G), ARAF (H), and CRAF (I). **(J)** Immunoblot analysis of 293T cells expressing BRAF (V600E), BRAF (V600E) CPD, and wild-type Myc-FBW7 and immunoprecipitated with an anti-BRAF antibody. Whole cell extracts were probed with the indicated antibodies. Data shown are representative of three independent and reproducible experiments. Respective proteins levels were quantified by ImageJ comparing indicated proteins to relevant controls.

To identify the role of DUBs in adaptive responses to MAPK pathway, we conducted an RNAi loss-of-function screen. Pools of shRNAs targeting 94 known or predicted DUBs were introduced into 293T cells and the abundance of phosphorylated ERK (pERK) to total ERK was quantified (Fig. S1 A and Table S1; Brummelkamp et al., 2003). After three rounds of selection, we identified nine shRNA pools that reproducibly showed a robust increase in the levels of pERK (USP28, UCH-L1, CYLD, USP49, USP19, TLI32, TRABID, USP42, and A20) and two shRNA pools that decreased levels of pERK (OTUD4 and BRCC36; Fig.1 A). Next, to determine whether expression of any of these genes are regulated by MAPK signaling in melanoma, we analyzed their expression by quantitative real-time PCR (qRT-PCR) after treatment with the BRAF inhibitor vemurafenib (PLX4032) in the BRAF (V600E) mutant melanoma cell line WM164. Vemurafenib treatment slightly increased the expression of USP28 and USP19 while inhibiting the expression of A20, CYLD, and UCHL1 (Fig. S1 B). We decided to focus our attention on USP28, as USP28 forms a complex with FBW7, a protein recently described to be mutated in melanoma (Cheng et al., 2013; Aydin et al., 2014). Furthermore, Western blot analysis indicated enhanced USP28 expression levels after vemurafenib treatment (Fig. 1 B). Importantly, USP28 expression was up-regulated after vemurafenib treatment in all of the BRAF (V600E) melanoma cell lines tested (Fig. S1 C).

Next, to confirm the validity of our RNAi screen we verified USP28 knockdown efficiency with our pooled USP28 shRNA constructs. To this end, we cotransfected 293T cells with the two hairpins isolated from the DUB pool (A and B) and two previously published hairpins (C and D; Popov et al., 2007). We observed that hairpins B, C, and D efficiently suppressed ectopically expressed and endogenous USP28 levels in 293T cells; however, hairpins C and D consistently achieved a greater knockdown efficiency than hairpins A and B and were therefore used for the remainder of the experiments. (Fig. 1 C; and Fig. S1, D and E). As expected, both shRNA vectors C and D efficiently enhanced the activation of ERK compared with controls (Fig. 1 D and Fig. S1 F). In contrast, ectopic expression of USP28, but not a catalytically inactive mutant, repressed phospho-ERK levels (Fig. 1 E). Collectively these results suggest that USP28 expression is regulated by MAPK activity and may function through a feedback loop to negatively regulate ERK signaling.

As USP28 is a component of the SCF ubiquitin ligase complex where it acts to stabilize FBW7, we hypothesized that loss of FBW7 would display comparable effects to USP28 knockdown on ERK activation. Indeed, knockdown of FBW7 by two validated shRNA vectors enhanced the levels of phosphorylated ERK, whereas in the presence of ectopically expressed BRAF, overexpression of FBW7 significantly decreased the levels of phosphorylated ERK (Fig. 1 F and Fig. S1, G and H). Importantly, BRAF expression was considerably diminished in the presence of FBW7 (Fig. 1 F).

Substrate recognition by FBW7 is regulated by phosphorylation within a conserved CDP (Cdc4 phosphodegron) motif of the substrate in which a central phosphothreonine is embedded within hydrophobic residues followed by a negative charge at the +4 position usually established through phosphorylation or the presence of glutamate (Fig. S2 A; Welcker and Clurman, 2008). This position makes direct contact with the WD40 repeats in FBW7, permitting substrate binding and recruitment of the SCF complex. We analyzed human RAF isoforms for CDP motifs and observed that both BRAF and ARAF have bona fide CPD domains, whereas CRAF contains a low-affinity degron lacking the +4 negative charge (Fig. S2 A). To confirm the interaction between FBW7 and RAF family members, we performed coimmunoprecipitation assays and found that FBW7 bound with high affinity to all three RAF isoforms (Fig. S2, B–D). Notably, ectopic expression of FBW7 decreased the expression of BRAF, ARAF, and CRAF (Fig. S2, B–D). Furthermore, ectopically expressed and endogenous USP28 coimmunoprecipitated with all three RAF isoforms (Fig.1, G–I; and Fig. S2, E–G). USP28 also interacted with FBW7 under physiological conditions, as seen by endogenous FBW7 coimmunoprecipitating with endogenous USP28 (Fig. S2 H). Because phosphorylation of the CDP motif is required for FBW7 substrate recognition, we sought to determine if BRAF is a direct target of the FBW7 complex. As shown in Fig. 1 J, site direct mutagenesis of both candidate phosphorylation sites within the Cdc4 phosphodegron motif to alanine (T403A and S408A) in BRAF, denoted as BRAF-CPD, decreased the association of FBW7 to mutant BRAF-CPD compared with its wild-type counterpart.

### USP28 regulates BRAF stability

**Figure 2.**
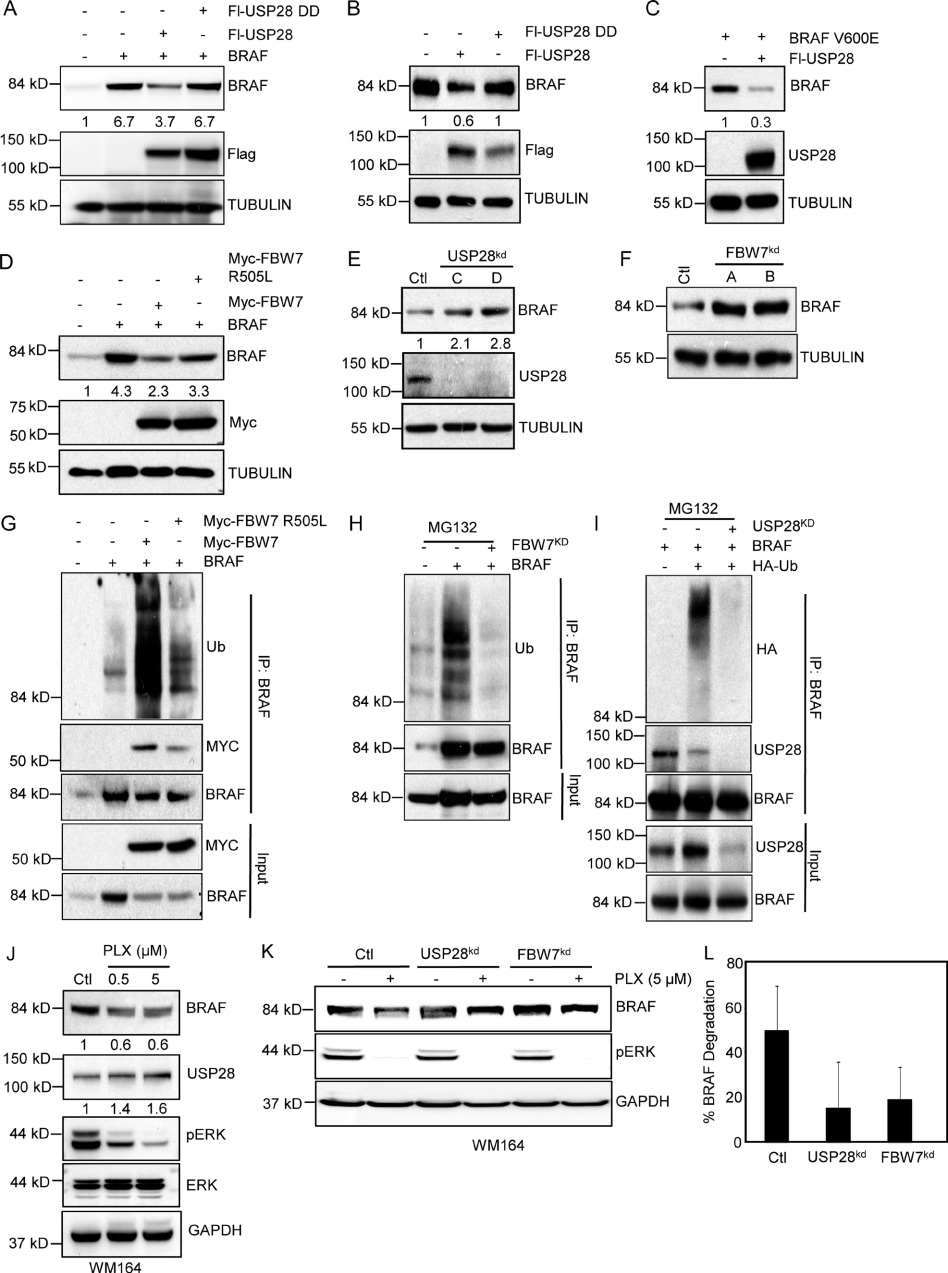
**USP28/FBW7 complex regulates BRAF stability. (A)** Representative images of immunoblot analysis of 293T cells expressing BRAF, Flag-USP28, or Flag-USP28 DD. Whole cell extracts were probed with the indicated antibodies. **(B)** Immunoblot analysis of 293T cells overexpressing Flag-USP28 or Flag-USP28 DD. Whole cell extracts were probed with the indicated antibodies. **(C)** Immunoblot analysis of 293T cells expressing mutant BRAF (V600E) and Flag-USP28. **(D)** Immunoblot analysis of 293T cells overexpressing BRAF, Myc-FBW7, or Myc-FBW7(R505L). Whole cell extracts were probed with the indicated antibodies. **(E)** Immunoblot analysis of 293T cells expressing shRNA vectors against USP28 (C and D). Whole cell extracts were probed with the indicated antibodies. **(F)** Immunoblot analysis in 293T cells expressing shRNA vectors targeting FBW7. **(G)** Immunoprecipitation with anti-BRAF resin in 293T cells overexpressing BRAF, Myc-FBW7, and Myc-FBW7 (R505L). Immunoblot analysis of indicated proteins is shown. **(H)** Immunoprecipitation with anti-BRAF resin in 293T cells expressing BRAF and an shRNA targeting FBW7 treated with proteasome inhibitor, MG132. Immunoblot analysis of indicated proteins is shown. **(I)** Immunoprecipitation with anti-BRAF in 293T cells expressing BRAF, shRNA vector targeting USP28, and HA-Ub, treated with proteasome inhibitor, MG132. Immunoblot analysis of indicated proteins is shown. **(J)** Immunoblot analysis of WM164 melanoma cells treated with PLX4032 at indicated concentrations for 18 h. Immunoblot analysis of indicated proteins is shown. **(K)** Immunoblot analysis of WM164 melanoma cells stably expressing shRNA vectors against USP28 or FBW7 and treated with PLX4032 (5 µM) for 18 h. Whole cell extracts were probed with the indicated antibodies. **(L)** Graph representing the percentage of BRAF degradation from three independent experiments after vemurafenib treatment as in K. Data shown are representative of three independent and reproducible experiments. Figure G, H, and I were performed in duplicate. Respective proteins levels were quantified by ImageJ comparing indicated proteins to relevant controls.

To limit unwanted FBW7 substrate degradation, FBW7 was autocatalytically ubiquitinated by the SCF complex resulting in its degradation. As USP28 deubiquitinates and stabilizes FBW7, allowing the FBW7/SCF ligase complex to bind and degrade substrates containing a Cdc4 phosphodegron motif, we hypothesized that forced expression of USP28 would target BRAF for degradation (Schülein-Völk et al., 2014). Indeed, overexpression of USP28 decreased the concentrations of ectopically expressed and endogenous BRAF levels (Fig. 2, A and B). This effect was dependent on the catalytic activity of USP28, as a USP28 inactive mutant did not have a major effect on either ectopic or endogenous BRAF stability (Fig. 2, A and B). Similar effects were observed with ectopic expression of USP28 and the hyperactive BRAF (V600E) mutant (Fig. 2 C). In agreement with previous results, overexpression of FBW7 diminished ectopically expressed BRAF (Fig. 2 D). However, this effect was nullified in cells transfected with the FBW7 WD40 domain mutant arginine 505 (R505L), which diminishes the ability of FBW7 to bind to the CDP motif in target proteins (Fig. 2 D). Using a FBW7 N-terminal antibody not present in the catalytic portion of our FBW7 construct, we determined that forced expression of FBW7 or FBW7(R505L) did not alter endogenous FBW7 levels nor alter the ability of endogenous FBW7 to dimerize with itself or form a complex with BRAF (Fig. S3 A). Having established that USP28 and FBW7 reduces BRAF stability, we tested the effect of USP28 and FBW7 depletion on BRAF expression. Both knockdown of USP28 and FBW7 significantly enhanced endogenous BRAF stability (Fig. 2, E and F). Next, to study the effect of FBW7/USP28 complex on BRAF ubiquitination, we cotransfected BRAF with either wild-type FBW7 or FBW7(R505L)-binding mutant and analyzed endogenous BRAF ubiquitination levels. As shown in Fig. 2 G FBW7 markedly enhanced BRAF ubiquitination, whereas FBW7(R505L) did not significantly alter the ubiquitination status of BRAF. Consistent with these results, suppression of either FBW7 or USP28, which would lead to the increased incorporation of ubiquitin and subsequent degradation of FBW7, by shRNA significantly inhibited the incorporation of ubiquitin into BRAF (Fig. 2, H and I). As USP28 potentially acts through a MAPK mediated feedback loop to regulate FBW7, we sought to address if vemurafenib altered FBW7 ubiquitination. Indeed, vemurafenib treatment decreased FBW7 ubiquitination (Fig. S3 B). Furthermore, vemurafenib treatment led to an overall decrease in BRAF stability (Fig. 2 J), an effect which was nullified in cells depleted for either USP28 or FBW7 (Fig. 2, K and L; and Fig. S3 C). However, we noted that loss of USP28 or FBW7 did not fully prevent BRAF degradation suggesting that BRAF degradation may occur through mechanisms independent of the USP28/FBW7 axis. Next, we sought to analyze if the increase in BRAF stability displayed in USP28-depleted cells correlated with up-regulation of MAPK activity after vemurafenib treatment. As expected USP28 mediated BRAF stability led to enhanced pERK levels after vemurfenib treatment compared with wild-type USP28 cells (Fig. S3 D). Collectively, these data demonstrate that BRAF ubiquitination and stability is directly regulated through an interplay between the FBW7/SCF ubiquitin ligase complex and the deubiquitinating enzyme USP28 leading to enhanced MAPK activity.

### USP28 is deleted in melanoma

**Figure 3.**
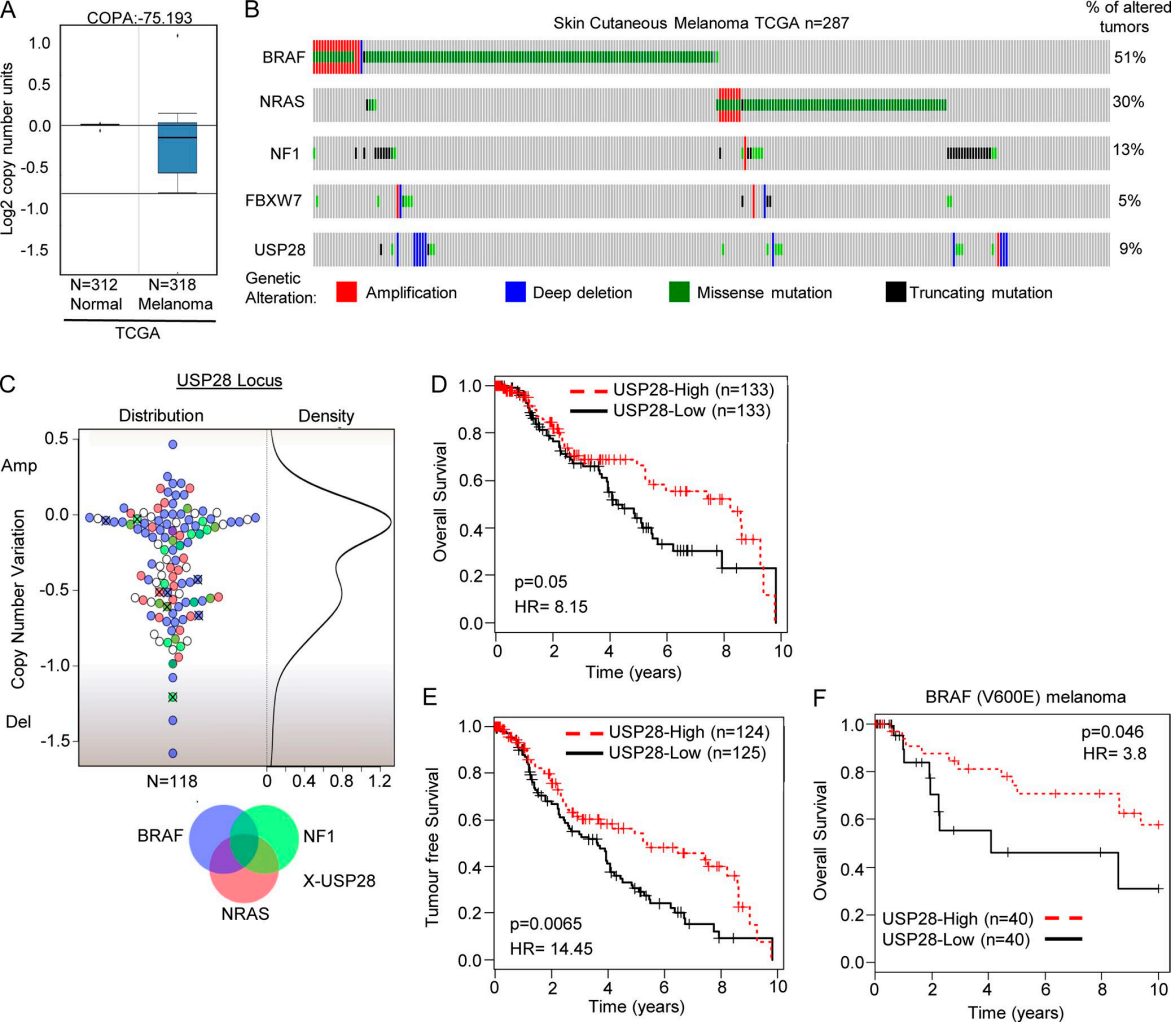
**USP28 is down-regulated in melanoma and confers poor prognosis. (A)** Oncomine box plot of USP28 in melanoma. **(B)** Matrix heat map generated using cBioportal showing genetic alterations of BRAF, NRAS, NF1, USP28, and FBW7 in melanoma patients (*n* = 287; TCGA). **(C)** Beeswarm plot demonstrating relative copy number variation of USP28 in melanoma patients, along with their respective mutational status of BRAF (blue), NRAS (red), NF1 (green), and USP28 (X) genes, respectively (*n* = 118). **(D)** Kaplan-Meier curves showing probability of overall survival of melanoma patients with lower copy number of USP28 is significantly less than those with higher level of USP28 (P = 0.05; HR = 8.15). **(E)** Kaplan-Meier curves showing probability of tumor free survival of melanoma patients with low levels of USP28 is significantly less than those with high levels of USP28 (P = 0.0065; HR = 14.45). **(F)** Kaplan-Meier survival analysis of melanoma patients harboring BRAF V600E mutation in respect to expression of USP28. Lower expression of USP28 confers poorer overall survival to melanoma patients carrying BRAF 600E mutation (P = 0.046; HR = 3.8).

Gain-of-function mutations within the MAPK pathway that lead to oncogenic activation of ERK are frequently found in several tumor types, including melanoma (Lito et al., 2013). Furthermore, it has been noted that in BRAF mutant melanoma, patient tumor responses are directly correlated with pERK down-regulation (Bollag et al., 2010; Spagnolo et al., 2014). Given the role of USP28 in the regulation of BRAF and pERK, we investigated the possibility that low USP28 might be a relevant factor in melanoma. In line with a study that FBW7 mutations have been observed in melanoma, oncomine expression analysis also revealed USP28 down-regulation in melanoma (Fig. 3 A, The Cancer Genome Atlas [TCGA]; Aydin et al., 2014). Next, we probed TCGA (cBioportal), where we observed that 9% of melanoma patients harbored mutations in USP28, with the majority of these mutations encompassing deletions of the gene (Fig. 3 B; Cerami et al., 2012). Similarly, analysis of COSMIC genome browser indicated a subset of melanoma patients containing focal deletions at the USP28 locus (unpublished data; Forbes et al., 2015). Interestingly, the frequency of coalterations between USP28 and FBW7 is low (0.05%; 2/39) indicating that nearly 13% (37/287) of all melanoma patients contain mutations within the FBW7–USP28 complex.

To further analyze the expression of USP28 in melanoma, we cross-referenced whole genome sequencing data with copy number variation (CNV) scores from 118 melanoma patients (Fig. 3 C and Table S2). USP28 expression was significantly down-regulated in 32% (38/118) of melanoma patients with 10% (11/118) of melanoma patients appearing to have only minimal USP28 expression. Importantly, of the 59 patients harboring BRAF V600E mutations 27% (16/59) displayed a >50% decrease in USP28 mRNA expression levels, suggesting that in tumors harboring BRAF alterations, loss of USP28 may further increase the tumorigenic potential of these tumors by stabilizing BRAF and enhancing downstream MAPK activation. To evaluate the clinical significance of USP28 in melanoma, we probed a publically available melanoma patient cohort (TCGA). This cohort contains 424 patients of which the disease stages are as follows: 1.4% for Stage 0, 18.2% for Stage I, 33.0% for Stage II, 39.9% for Stage III, 5.2% for Stage IV, and 2.3% for Stage I/II not otherwise specified (NOS; Fig. S4 A). Stratification of patients into two groups based on the expression of USP28 determined that patients with low expression of USP28 had significantly reduced survival and reduced tumor-free survival (Fig. 3, D and E). Furthermore, MANOVA analysis determined USP28 as an independent prognostic factor for survival (P = 5.88 × 10^−6^; Fig. S4 B). Cross-correlation of USP28 expression in BRAF (V600E) melanoma patients indicated that in this subset of patients, once again low levels of USP28 conferred lower overall survival (Fig. 3 F). Collectively these results indicate that USP28 is frequently mutated in melanoma and that low expression levels of USP28 correlate with poor overall survival.

### Loss of USP28 enhances BRAF stabilization and confers resistance to vemurafenib in melanoma

**Figure 4.**
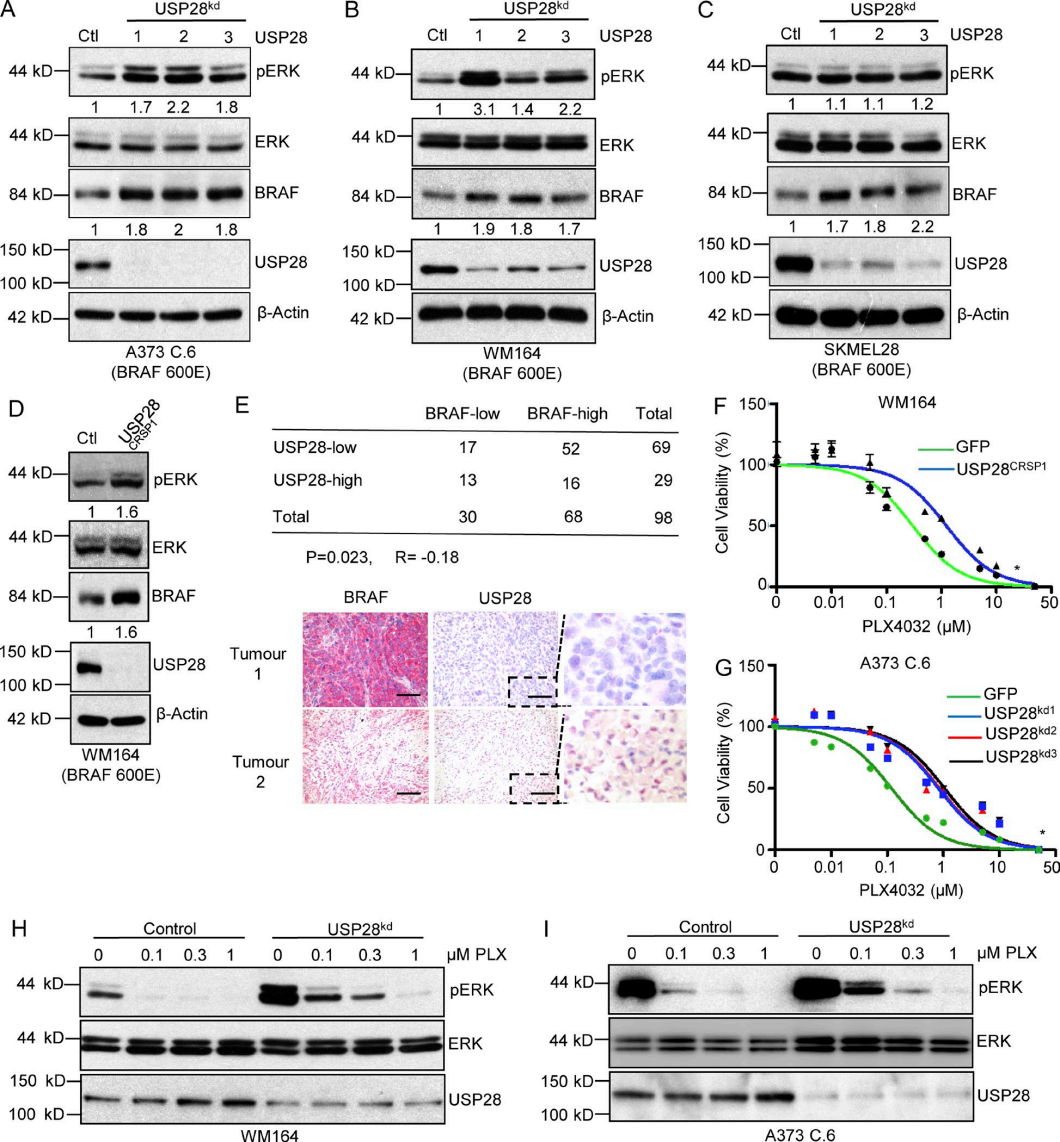
**Down-regulation of USP28 leads to BRAF inhibitor resistance. (A–C)** Representative images of immunoblot analysis of BRAF (V600E) mutant melanoma cell lines A373C.6 (A), WM164 (B), and SK-MEL-28 (C) infected with scrambled or USP28 shRNA lentivirus. Whole cell extracts were probed with the indicated antibodies. Data shown are representative of three independent and reproducible experiments. **(D)** Immunoblot analysis of WM164 or WM164^USP28CRSP^ cells. Whole cell extracts were probed with the indicated antibodies. Data shown are representative of three independent and reproducible experiments. **(E)** Correlation between USP28 and BRAF protein levels in melanoma patients (*n* = 98). Statistical significance was determined by an χ^2^ test (P = 0.023). R is the correlation coefficient (R=−0.18; top). Immunohistochemical staining of BRAF and USP28 on sequential sections of ME2082B (Biomax) melanoma tissue microarray. Red staining indicates positive immunoreactivity. Bars, 50 µm. Dashed boxes indicate zoomed area. **(F)** WM164 or WM164^USP28CRSP^ cells treated with escalating doses of vemurafenib (PLX4032) for 72 h. Viability was assessed using CellTiter Glo as described by the manufacturer. Data represent the mean of six replicates. **(G)** A373C.6 cells or A373C.6 USP28 knockdown cells treated with escalating doses of vemurafenib (PLX4032) for 72 h. Viability was assessed using CellTiter Glo as described by the manufacturer. Data represent the mean of six replicates. **(H)** Immunoblot analysis of WM164 or WM164 USP28 knockdown cells treated with different concentrations of vemurafenib (PLX4032) for 1 h. Whole cell extracts were probed with the indicated antibodies. Data shown are representative of three independent and reproducible experiments. **(I)** Immunoblot analysis of A373C.6 or A373C.6 USP28 knockdown cells treated with different concentrations of vemurafenib (PLX4032) for 1 h. Whole cell extracts were probed with the indicated antibodies. Data shown are representative of three independent and reproducible experiments. For respective immunoblots proteins levels were quantified by ImageJ comparing indicated proteins to relevant controls.

Because USP28 is frequently deleted in melanoma and USP28 depletion leads to BRAF stability and enhanced MAPK kinase in HEK293T cells, we asked if interfering with USP28 expression conferred a similar response in BRAF (V600E) melanoma cell lines. In line with previous results, depletion of USP28 in all the melanoma cell lines tested resulted in increased stabilization of BRAF and enhanced downstream MAPK activation (Fig. 4, A–C). One exception was observed in SK-MEL-28 cells, where knockdown of USP28 demonstrated BRAF stabilization but not complementary pERK activation, indicating that downstream BRAF signaling may not be limiting factor for ERK phosphorylation in these cells (Fig. 4 C). Moreover, generation of WM164 USP28 knockout cells using the CRISPR/CAS9 endonuclease displayed similar intercellular responses as USP28 knockdown cells (Fig. 4 D).

In light of these observations, we sought to establish whether USP28 expression was inversely correlated with BRAF expression in melanoma patients. Immunohistochemical staining of USP28 and BRAF was performed on melanoma tissue microarrays containing cores from 98 individual primary melanomas. Notably, down-regulation of USP28 correlated with high BRAF levels in 75.4% (52/69) of melanoma tumors compared with 55% (16/29) of tumors which displayed both high USP28 and high BRAF (P = 0.023 and R= −0.18; Fig. 4 E). However, no direct relationship was observed in tumors expressing low levels of BRAF with overall USP28 expression. These findings suggest that loss of USP28 contributes to a significant up-regulation of BRAF in a substantial fraction of melanoma patients.

As hyperactivation of the MAPK pathway has previously been demonstrated to enhance resistance to MAPK pathway inhibitors, we speculated that loss of USP28 in BRAF (V600E) melanoma cell lines would limit sensitivity of these cells lines to the BRAF inhibitor vemurafenib (Corcoran et al., 2010). As anticipated, USP28-depleted melanoma cell lines were more resistant to BRAF inhibitor treatment and then their wild-type counterparts, as demonstrated by a rightward shift in the dose–response curve (Fig. 4, F and G; Fig. S5, A and B). Consistent with this finding, vemurafenib resistance in USP28 knockdown cell lines was associated with sustained ERK phosphorylation (Fig. 4, H and I; and Fig. S5, C and D). It is important to note that in both cell lines tested vemurafenib treatment enhanced the expression USP28, indicative of a USP28 feedback loop in both these BRAF mutant cell lines (Fig. 4, I and J). These results suggest that loss of USP28 regulates the sensitivity of melanoma cells to BRAF inhibition through hyperactivation of the MAPK pathway and downstream ERK signaling.

**Figure 5.**
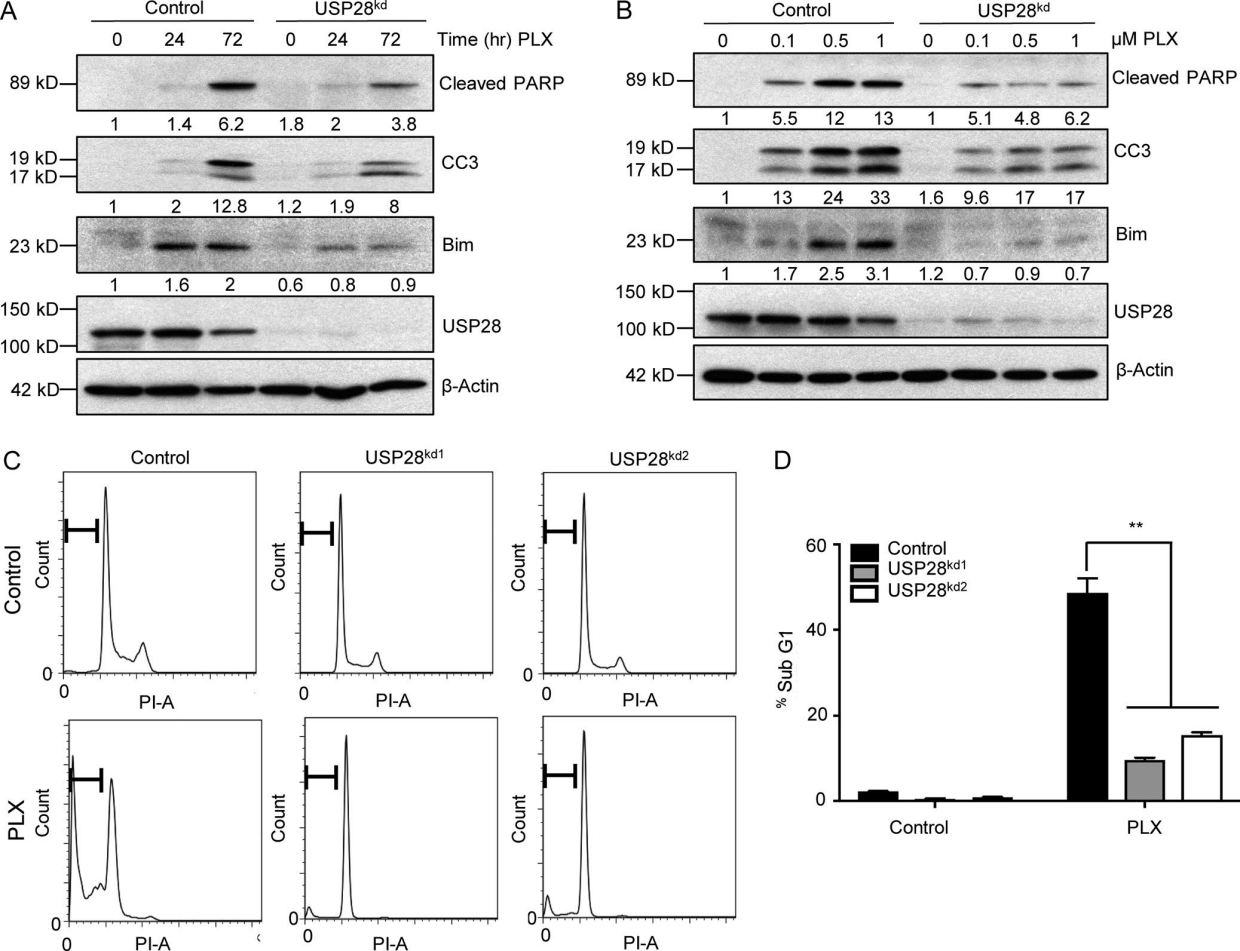
**Down-regulation of USP28 impairs apoptosis induced by vemurafenib. (A)** Representative images of immunoblot analysis of A373C.6 or A373C.6 USP28 knockdown cells treated with 2 µM vemurafenib (PLX4032) for indicated time points. Whole cell extracts were probed with the indicated antibodies. Data shown are representative of two independent and reproducible experiments. Respective proteins levels were quantified by ImageJ comparing indicated proteins to relevant controls. **(B)** Representative images of immunoblot analysis of A373C.6 or A373C.6 USP28 knockdown cells treated with different concentrations of vemurafenib (PLX4032) for 72 h. Whole cell extracts were probed with the indicated antibodies. Data shown are representative of two independent and reproducible experiments. Respective proteins levels were quantified by ImageJ comparing indicated proteins to relevant controls. **(C)** Representative images of cell-cycle analysis of A373C.6 or A373C.6 USP28 knockdown cells after 72 h of treatment with vemurafenib (2 µM). Data shown are representative of three independent and reproducible experiments. **(D)** Quantification of sub-G_1_ population after treatment with vemurafenib as indicated, mean ± SEM of three independent experiments. A two-tailed Student’s *t* test compares the treated populations; **, P < 0.01.

Next, we sought to study the differences in cellular responses as a result of enhanced MAPK pathway activation in USP28-depleted cells. As USP28 knockdown inhibited the ability of vemurafenib to attenuate ERK phosphorylation, we reasoned that in these cell lines BIM (Bcl-2–interacting mediator of cell death) accumulation would be down-regulated. BIM is negatively regulated by ERK kinase though direct phosphorylation, targeting BIM for proteasomal mediated degradation. The up-regulation of BIM has been implicated as an essential factor in the induction of apoptosis after MAPK pathway inhibition (Wickenden et al., 2008). The addition of vemurafenib substantially stabilized BIM resulting in enhanced PARP and caspase 3 cleavage, indicative of the induction of apoptosis (Fig. 5 A). In contrast, in USP28-depleted cells BIM, cleaved PARP, and cleaved caspase 3 levels were markedly reduced (Fig. 5 A). Moreover, treatment with vemurafenib enhanced the expression of these apoptotic markers in a dose-dependent manner in control cells, an effect which was once again attenuated in USP28-depleted cells (Fig. 5 B). USP28-depleted cells also exhibited a decrease in the accumulation of cells in Sub-G_1_, compared with control cells after vemurafenib treatment (Fig. 5, C and D). These data suggest that continued activation of ERK signaling and inhibition of apoptosis may at least in part play a critical role in vemurafenib resistance in cells with repressed USP28 expression.

### USP28 mediates vemurafenib sensitivity in vivo

**Figure 6.**
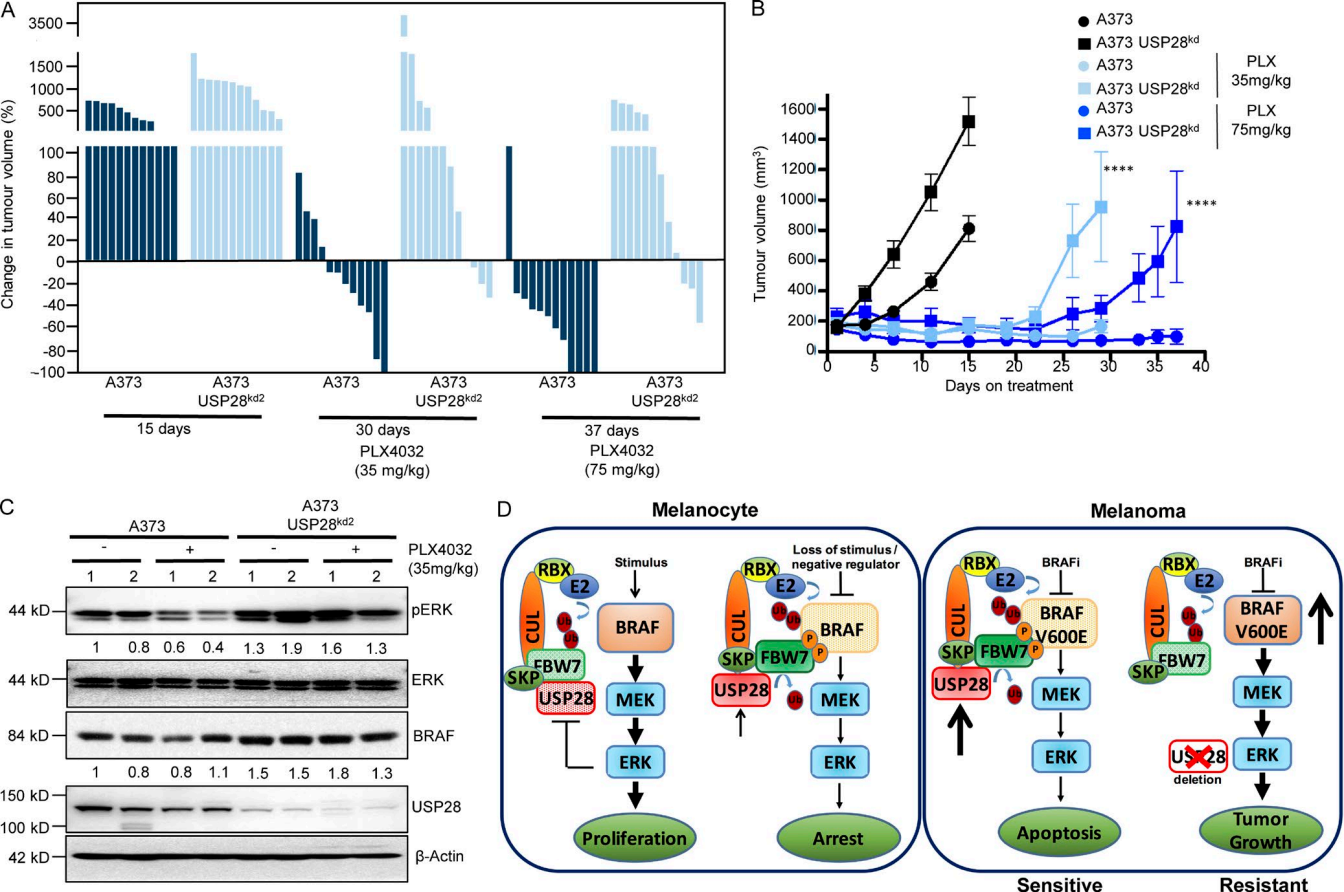
**Down-regulation of *USP28* impairs the effects of vemurafenib in vivo. (A)** Waterfall plot showing the percentage change in tumor volume for the individual tumors at day 15 for untreated controls, day 30 for mice treated twice daily with 35 mg/kg and day 37 for mice treated with 75 mg/kg (*n* = 12). **(B)** Quantification of nude mice bearing xenograft tumors of A373 C.6 or A373 C.6 USP28 knockdown cells (*n* = 12). Mice were treated twice daily with vemurafenib (PLX4032; 35 mg/kg, light blue) for 30 d or (75 mg/kg, dark blue) for 37 d (end of experiment). Points indicate mean tumor volume; bars, SE. A two-tailed Student’s *t* test compares the two treated grouped populations of control cells versus USP28-depleted cells. ****, P < 0.0001. **(C)** Immunoblot analysis of mouse xenograft A373 C.6 melanoma tumors stably infected with USP28 knockdown vector or relevant controls. Tumor lysates were analyzed with indicated antibodies. Data shown are representative of two independent and reproducible experiments. 1 and 2 indicate two individual mice. Respective proteins levels were quantified by ImageJ comparing indicated proteins to relevant controls. **(D)** Schematic of vemurafenib resistance in USP28 deleted melanoma cells.

Next, we sought to determine whether loss of USP28 regulated vemurafenib resistance in vivo. To this end, we injected immunodeficient mice with A373-C6 cells stably depleted for USP28 or shRNA control counterparts. Vemurafenib treatment was started 7 d after injection, when the tumors reached a volume of 200 mm^3^. Vemurafenib treatment was dosed at either 35 mg/kg or 75 mg/kg. Silencing of USP28 expression in A373-C6 xenografts significantly decreased sensitivity to vemurafenib-induced tumor shrinkage at both concentrations tested, compared with control mice (Fig. 6, A and B). Pharmacodynamics studies demonstrated that depletion of USP28 led to a robust retention of ERK phosphorylation in tumors treated with vemurafenib (Fig. 6 C). Furthermore, all USP28 knockdown tumors displayed increased stabilization of BRAF (Fig. 6 C). Collectively these results suggest that down-regulation of USP28 decreases BRAF inhibitor sensitivity in vivo.

We next assessed whether USP28 expression was inversely correlated with response to RAF and MEK inhibition in patients with mutant BRAF. We analyzed USP28 expression by immunohistochemistry (IHC; H-score) and compared overall time to progression in BRAF (V600E) patients treated with combined RAF and MEK inhibitor treatment (dabrafenib and trametinib or vemurafenib and combimetinib; *n* = 5). Patients’ prognosis of progressive disease, partial response, or complete response was determined by RECIST (response evaluation criteria in solid tumors). Mean H-score was determined as a cutoff for patients exhibiting high USP28 (H-score > 100) or low USP28 (H-score < 100). Importantly, low USP28-expressing tumors exhibited a shorter time to progression than tumors with high USP28 (Fig. S5 E). However, it is important to note that this analysis is based on a small sample size and is required to be verified in a larger cohort (*n* = 5). Collectively our results show that loss of USP28 enhances downstream MAPK activation through the stabilization of BRAF, leading to decreased sensitivity to combination therapies involving BRAF inhibitors dabrafenib or vemurafenib (Fig. 6 D).

### Rigosertib is synthetically lethal with USP28 loss

**Figure 7.**
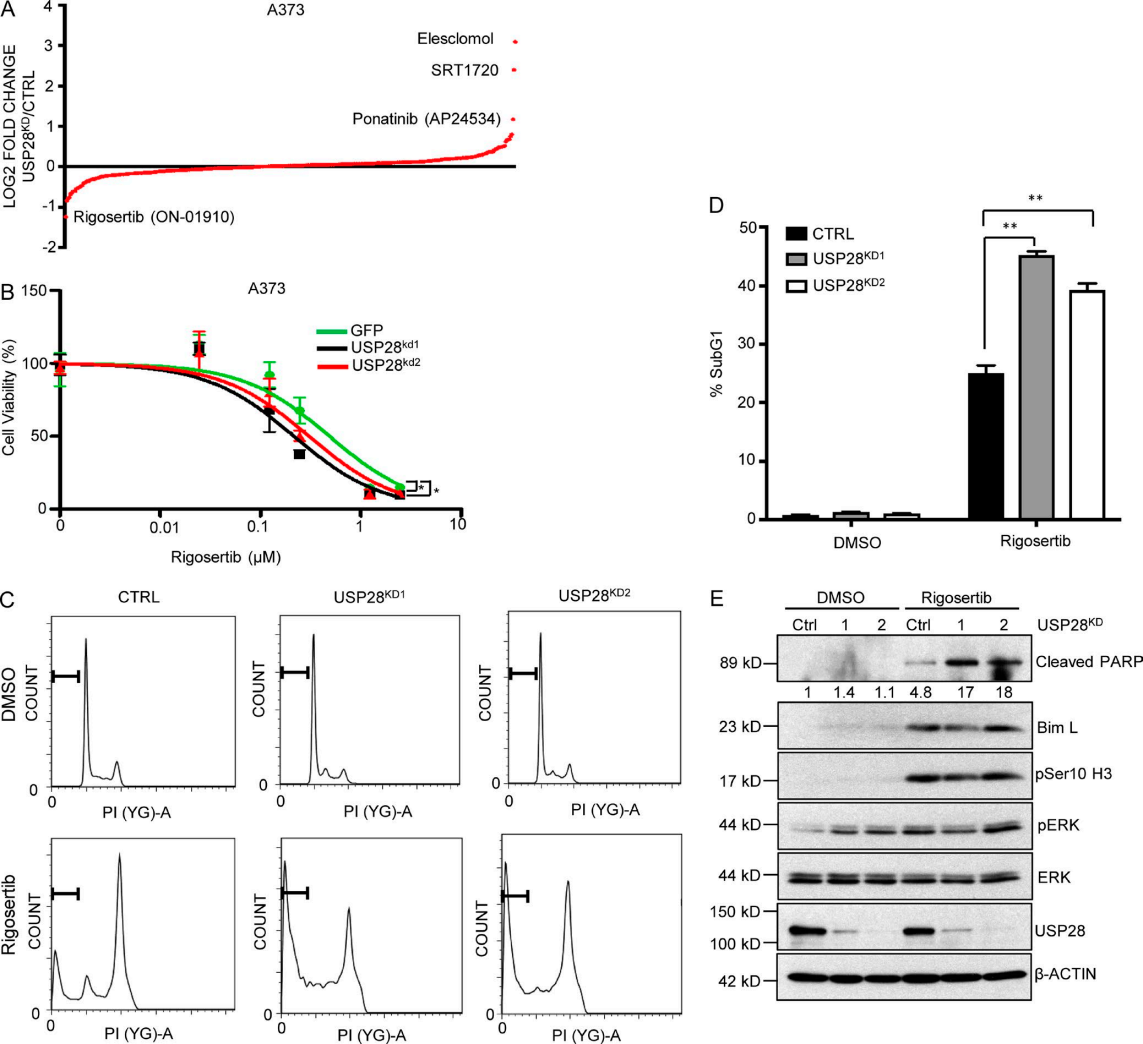
**Selective synthetic lethality with Rigosertib to USP28 loss. (A)** Mean rank-order plot demonstrating fold change from FDA-approved chemical compound screen (316 compounds) in A373C.6 cells versus A373C.6 USP28 knockdown cells. Data represent the mean of three replicates. **(B)** A373C.6 cells or A373C.6 USP28 knockdown cells treated with escalating doses of rigosertib for 72 h. Viability was assessed using Cell-Titer Glo as described by the manufacturer. Data represent the mean of six replicates. A373 GFP versus A373 USP^kd1^; *, P < 0.0001 (nonlinear regression, extra sum-of-squares test). A373 GFP versus A373 USP^kd2^; *, P < 0.001 (nonlinear regression, extra sum-of-squares test). **(C)** Representative images of cell-cycle analysis of A373C.6 or A373C.6 USP28 knockdown cells after 48 h of treatment with rigosertib (300 nM). Data shown are representative of three independent and reproducible experiments. **(D)** Quantification of sub-G_1_ population after treatment with rigosertib as indicated, mean ± SEM of three independent experiments. A two-tailed Student’s *t* test compares the treated populations; **, P < 0.01. **(E)** Immunoblot analysis of A373C.6 or A373C.6 USP28 knockdown cells treated with rigosertib (300 µM) for 48 h. Whole cell extracts were probed with the indicated antibodies. Data shown are representative of three independent and reproducible experiments. Respective proteins levels were quantified by ImageJ comparing indicated proteins to relevant controls.

To search for synthetic lethal interactions in melanoma cell lines depleted for USP28, we performed a high throughput synthetic lethal chemical compound screen. Using an ATP-based cell viability assay (Cell Titer-Glo), we screened a small library of 316 FDA-approved chemical compounds and identified the PLK1 inhibitor rigosertib as a compound that selectively impairs the viability of USP28-depleted cells (Fig. 7 A and Table S3). To corroborate the sensitivity of USP28-depleted melanoma cells to rigosertib, we analyzed cell viability in a dose-dependent manner. Once again USP28-depleted cells were more sensitive to rigosertib than control cells, as demonstrated by a leftward shift in a dose response curve (Fig. 7 B and Fig. S5 F). It is important to note that USP28 also rendered cells resistant to SRT1720, elesclomol, and ponatinib, the latter two of which are presently explored in late-phase clinical trials for various cancers (Fig. 7 A). Rigosertib is a styrylbenzyl sulfone that acts as a non-ATP competitive inhibitor of polo-like kinase (PLK1) and phosphinositide 3-kinase (PI3K), inducing mitotic arrest and apoptosis (Okabe et al., 2015). However, rigosertib has also recently been identified as a RAS-mimetic, interacting with RAS-binding domains of RAF kinases perturbing RAS-RAF binding (Athuluri-Divakar et al., 2016). Apart from activating downstream MAPK activation, CRAF also plays a MEK independent role in regulating mitosis and tumor progression. After RAS activation, CRAF becomes phosphorylated at S338, inducing CRAF localization at the mitotic spindles and complex formation with PLK1 and Aurora kinase A during G2/M phase of the cell cycle permitting cell cycle progression (Mielgo et al., 2011). Conversely, inhibition of RAF phosphorylation impairs RAF-PLK1 interaction and PLK1 activation, inducing prometaphase arrest and apoptosis (Athuluri-Divakar et al., 2016). It is a phenotype equally observed in RAS mutant cell lines after the exposure of PLK1 inhibitors (Luo et al., 2009). To examine if the effect in the delay of mitotic exit resulted in enhanced apoptosis, we examined the Sub-G_1_ fraction of cells 48 h after rigosertib treatment. As expected, rigosertib treatment potently induced a G2/M arrest in A373 cells (Fig. 7 C). In addition, USP28-depleted cells exhibited a significant increase in the accumulation of cells in Sub-G_1_ compared with control cells after rigosertib treatment. (Fig. 7, C and D). In line with these results, USP28-depleted cells demonstrated a robust increase in cleaved PARP compared with their parental counterparts (Fig. 7 E). Interestingly, rigosertib only induced ERK phosphorylation in USP28 expressing cells, bringing overall phospho-ERK levels even with USP28-depleted cells resulting in no overall change to BIM stability following rigosertib treatment. This indicates that the enhanced apoptosis observed in USP28 knockdown cells exposed to rigosertib may be ERK independent. Future work will be required to elucidate the mechanism of rigosertib sensitivity in USP28 knockdown cells. Collectively, these data suggest that rigosertib specifically enhances apoptosis in USP28-depleted cells.

## Discussion

The administration of targeted therapies in patients with defined tumor-driving lesions have proven to function as effective anticancer agents. For example, selective RAF inhibitors such as vemurafenib have demonstrated clinical efficacy in BRAF V600E mutant melanomas. However, overall response rates to these therapies remain disappointing with quantifiable tumor regression over time limited by mechanisms of intrinsic and acquired resistance. Interestingly, in the majority of these cases incomplete pERK down-regulation has been associated with resistance to MAPK pathway inhibitors (Bollag et al., 2010; Lito et al., 2013; Morris et al., 2013). One of the mechanisms through which this occurs is through the loss of negative feedback inhibition on upstream components of the MAPK pathway after treatment with MAPK pathway inhibitors. Therefore, a deep understanding of the intercellular signaling pathways and dependent feedback mechanisms involved in MAPK signaling and the identification of reliable biomarkers is critical in determining how these targeted agents may elicit prolonged responses in patients. Using a functional RNAi screen followed by transcriptomic analysis, we identify a novel ubiquitin mediated adaptive response in the regulation of MAPK signaling. In melanoma cell lines, USP28 expression is positively regulated after MAPK inhibition, whereby USP28 enhances down-regulation of the MAPK pathway through SCF mediated ligase degradation of RAF family members.

In this setting, USP28 deubiquitinates the SCF component FBW7, allowing FBW7 to act as a substrate recognition factor targeting substrates for proteosomal mediated degradation. FBW7 proteins consist of three functional domains, one of which, the WD40 domain, is critical for the recognition of specific phosphodegron motifs on target proteins. Recently, whole exome sequencing in a cohort of 77 melanoma samples identified several recurrent mutations within the WD40 domain of FBW7, suggesting that loss of effective substrate recognition by FBW7 may limit protein turnover of SCF complex targets (Aydin et al., 2014). FBW7 is considered a tumor suppressor targeting several dominant oncogenes such as c-MYC, CYCLIN E, c-JUN, and NOTCH for proteasomal degradation. It is therefore unsurprising that FBW7 mutations have been described in several human neoplasias (Akhoondi et al., 2007; Welcker and Clurman, 2008). Here, we identify RAF family members as a FBW7 substrate in melanoma.

Interestingly, down-regulation of several components of the SCF ligase complex have previously been demonstrated to limit sensitivity to BRAF inhibition in melanoma cell lines. Loss of either CUL3, FBXL6, or RBX1 expression conferred a growth advantage in the presence of vemurafenib, indicating that an unimpaired SCF complex is critical for vemurafenib sensitivity in melanoma (Shalem et al., 2014; Kim et al., 2015). Similarly, we observe a link between depletion of USP28 and BRAF inhibitor resistance in vitro and in vivo (Fig. 6 E). Furthermore, our initial assessment on a cohort of patient tumors treated with a combination of BRAF and MEK inhibitors indicates that the USP28/FBW7 regulatory axis is a relevant determinant for sustained patient responses, an observation that requires confirmation in larger patient cohorts.

In an attempt to find a way to overcome USP28-mediated BRAF inhibitor resistance, we performed a synthetic lethal screen using a library of FDA-approved small molecules. Our screen identified rigosertib, a known PLK1 inhibitor and RAS mimetic, to induce apoptosis to a greater extent in USP28-depleted cells compared with parental cells. We demonstrate that USP28-depleted cells exhibit higher levels of cleaved PARP, resulting in a corresponding increase in apoptosis. Although our results also showed a higher level of BIM in rigosertib-treated cells, no change in BIM levels was observed between USP28-depleted and control cells. This result is consistent with our observation that no change was detected in levels of phospho-ERK between USP28-depleted cells and control cells after rigosertib treatment. These results indicate that rigosertib-induced apoptosis in USP28-depleted cells may be a result of a MAPK-BIM–independent pathway. Interestingly, it has been shown that rigosertib, similar to other compounds, such as taxol, KG5, nocodazol, and vincristine, is able to induce mitotic stress, JNK-dependent apoptosis, and microtubule disruption (Mäki-Jouppila et al., 2014; Prasad et al., 2016; Ritt et al., 2016). Microtubule disruption is widely known to lead to CASPASE-2–mediated apoptosis (Ho et al., 2008). Dimerized active CASPASE-2 causes apoptosis in a BID dependent but not a BIM-dependent manner (Mahajan et al., 2014). These observations are in parallel with our results in synthetic lethal screens, where we observed that USP28-depleted cells are more sensitive to small molecules (rigosertib, BI-2536, nocodazole, and vincristine; Table S3) with the ability to disrupt the cytoskeleton. This suggests a potential role for USP28 in the mitotic spindle assembly complex. The combination of these results stresses the need for further investigation to determine the precise mechanism of rigosertib specifically targeting USP28 mutant cells.

Collectively, our findings unveil a novel ubiquitin-mediated feedback loop in the regulation of BRAF family members after BRAF inhibition. An adaptive response that is lost in melanoma patients harboring mutations in USP28 resulting in BRAF stabilization, hyperactivation of the MAPK signaling, and resistance to therapies targeting this pathway. Furthermore, we demonstrate that cells exhibiting USP28 loss are synthetically lethal with the RAF-PLK1 inhibitor rigosertib. Our findings uncover a new mechanistic biomarker for resistance in patients receiving RAF and MEK inhibitors in the clinic. Moreover, we identify a new promising therapeutic strategy to potentially enhance survival in patients with USP28 mutations.

## Materials and methods

### TCGA data analysis and patient stratification

For beeswarm plot, CNV scores were derived from the level 3 “CNV Low Pass DNASeq” analysis of TCGA HMS Illumina HiSeqDNASeqC. Somatic mutations were derived from the level 2 (maf files) from the TCGA BI Illumina GA DNASeq. For Kaplan Meir analysis, we used a publicly available dataset for survival data analysis of melanoma tumor patients with respect to USP28 expression. The public patient data were the RNAseq data downloaded from TCGA (Melanoma patient cohort). This cohort contains 424 patients of which the disease stages are as follows: 1.4% for Stage 0, 18.2% for Stage I, 33.0% for Stage II, 39.9% for Stage III, 5.2% for Stage IV, and 2.3% for Stage I/II NOS. Before survival analysis, raw RNAseq counts were normalized using the total numbers of mappable reads across all samples. Normalized USP28 expression data were then used for survival analysis. In the survival analysis, the top and bottom thirds of USP28 expression groups across all patient samples were used to define USP28^high^ and USP28^low^ patient groups. The Kaplan-Meier method was then applied for depicting the survival difference between the USP28^high^ and USP28^low^ groups, and the log-rank test was used for determining the statistical significance. Similarly, we applied the above approach to a subset of this cohort of melanoma patients harboring BRAF V600E mutation in survival analysis using USP28 expression as a marker.

### qRT-PCR

Cells were collected, washed twice in PBS and RNA was isolated using GeneJet RNA extraction kit (Thermo Scientific) and BIOER Total RNA extraction kit (BSC52M1). qRT-PCR was performed using SYBR green from Applied Biosystems according to manufacturer’s recommendations. Reactions were performed on an ABI 7500 FAST instrument. Relative mRNA values were calculated by the ΔΔCt method. GAPDH were used as internal normalization controls where specified. The following QRT primers were used: USP28, 5′-ACTCAGACTATTGAACAGATGTACTGC-3′ and 5′-CTGCATGCAAGCGATAAGG-3′; GAPDH, 5′-CATACCAGGAAATGAGCTTGAC-3′ and 5′-AACAGCGACACCCACTCCTC -3′; A20, 5′-GTGGCCTTTTGTGATGGTTT-3′ and 5′-GCTTTTGCTGTCCCAATACC-3′; USP42, 5′-GCTCGACGGATGAAATGAGT-3′ and 5′-CTGGCTCCTCCAGGGATT-3′; USP32P, 5′-CCTCTGCTGCTCATAGAAAAGAA-3′ and 5′-ACAATGGCAGCATCTGTGAG-3′; ZRNAIB1, 5′-TGGCTATACTCTTGTACACTTGGCTA-3′ and 5′-TGCTGCTTGTTGAGACACCT-3′; CYLD, 5′-GGGTAGCCCCCTACTGTTCT-3′ and 5′-CCCCAACTATGTGCCTCTTG-3′; USP19, 5′-GGCACCGGCAGATAAAGAAA-3′ and 5′-CGGCACAAGATGAGGGA-3′; UCHL-1, 5′-AGATCAACCCCGAGATGCT-3′ and 5′-ACCGAGCCCAGAGACTCC-3′.

### Cell culture, transfection and immunoblotting

HEK293T cells and all melanoma cells (WM164, A2058, A373 C.6, and SK-MEL-28) were maintained in standard DMEM (Gibco) supplemented with 4.5 g/liter d-Glucose and l-Glutamine and 110 mg/liter Sodium Pyruvate. The media was further supplemented with 10% FBS (Gibco) and 1% antibiotics (penicillin/streptomycin; Gibco). Cells were grown in a 5% CO_2_ atmosphere at 37°C. Transfection of HEK293T cells was done using calcium chloride and Hepes buffered saline, pH 6.95. 16 h after transfection, media were aspirated, and cells were washed twice with 1× PBS and replenished with fresh media. 24 h later, cells were washed twice with 1× PBS and lysed with RIPA lysis buffer (50 mM Tris-HCl, pH 7.4, 150 mM NaCl, 1% Nonidet P-40, 0.1% SDS, and 0.5% sodium deoxycholate) supplemented with Protease inhibitor cocktail (Complete EDTA-free tablet; Roche) and phosphatase inhibitors (50 mM sodium fluoride, 10 mM β-glycerophosphate, 1 mM magnesium chloride, and 1 mM sodium orthovanadate). The cells were lysed and kept on ice for 30 min before spinning down at 12,000 rpm for 15 min, supernatants were transferred to new tubes, and protein estimation was done using BCA Protein estimation kit (Thermo Scientific); 30 µg of lysates were boiled in sample buffer containing 10% β-mercaptoethanol and loaded onto a 10% Acrylamide SDS-PAGE gel. Immunoblotting was done on a polyvinylidene difluoride membrane using wet transfer method. The membrane was blocked with BSA for an hour at room temperature before probing it with the appropriate primary antibody overnight in 4°C. The membranes were washed three times with TBS (Tris-buffered saline)–0.1% Tween 20 before incubation with proper secondary antibodies for an hour. The membranes were washed three times with TBS–0.1% Tween 20 and visualized using ECL reagent from Thermo Scientific or Amersham (GE). To detect endogenous ubiquitin, blots were treated and probed as described by Penengo et al. (Penengo et al., 2006) All immunoblotting experiments were performed in triplicate unless otherwise indicated. Western blots were quantified using ImageJ (National Institutes of Health) with relative phosphorylated ERK levels calculated as a percentage of total ERK protein (pERK/ERK), and relative BRAF levels were calculated as a percentage of appropriate loading control (BRAF/β-Actin).

### Immunoprecipitation and in vivo deubiquitination assay

For coimmunoprecipitation experiments, cells were lysed in ELB (0.25 M NaCL, 0.1% NP-40, and 50mM Hepes, pH 7.3) supplemented with proteasome inhibitors. Cell lystates (500 µg–1 mg) were incubated for 2 h to overnight with 2 µg of the indicated antibodies conjugated to protein A or protein G sepharose beads (GE Healthcare), washed three times in ELB buffer, and separated out on SDS-PAGE gels. In vivo deubiquitination experiments were performed as in Kit et al. (Kit Leng Lui et al., 2017). In brief, BRAF (5 µg) alone or along with USP28 shRNA (20 µg) was cotransfected with HA-Ubiquitin (5 µg) or a control vector. For endogenous ubiquitination experiments, BRAF (5 µg) was cotransfected with Myc-tagged FBW7 (5 µg) or Myc-tagged FBW7 mutant (5 µg) or FBW7 shRNA (20 µg) or control vectors. After 72 h MG132 (5 µM) was added, incubated overnight, and cells were lysed in ELB buffer. The level of ubiquitination was measured using Ubiquitin antibody (Santa Cruz) or HA antibody.

### Plasmids, reagents, and antibodies

The DUB knockdown library vectors were generated by annealing the individual oligonucleotide primer pairs and cloning them into pSuper as described in Brummelkamp et al. (2003). The bacterial colonies of each DUB hairpin were then pooled and used for plasmid preparation. For USP28 knockdown, pRetrosuper vectors targeting the following sequences were used: (A) 5′-GGAAAGTACCAAGAGGCAC-3′; (B) 5′-GTACAAGTACAGAAAGCT-3′; (C) 5′-GGAGTGAGATTGAACAAGA-3′; and (D) 5′-GTATGGACAAGAGCGTTGGT-3′. Lentiviral knockdown vectors targeting USP28 were purchased from Transomic. Lentiviral sequences are as follows: (1) 5′-TTCGGAACAAACTATAATCTTC-3′; (2) 5′-TTGTGATGTAGAGTAGTCCTGT-3′; (3) 5′-TTAGCTAAGATTTTTATCTGCA-3′. The lentiviral vector with nucleotide sequence of 5′-ATGCTTTGCATACTTCTGCCTG-3′ were used as control. Lentiviral knockdown vectors targeting FBW7 were purchased from Darmacon. Lentiviral sequences are as follows: (a) 5′-ATTCCACTTGTTAACGACTGG-3′ and (b) 5′-TAGACAGGTTTCAGTCTCTGG-3′. FLAG-tagged BRAF 600E construct containing CPD (T-A and S-A) mutants was generated using site directed mutagenesis. To perform the reaction, following primers were used: 5′-TTTGTCTGCTGCCCCCCCTGCCGCATTACCT-3′ and 5′-AGGTAATGCGGCAGGGGGGGCAGCAGACAAA-3′. Myc-tagged FBW7 R505L mutant was generated by site directed mutagenesis using the following primers: 5′-CAGCAGTCCTCTGTGTTCAAT-3′ and 5′-ATTGAACACAGAGGACTGCTG-3′. Flag-tagged USP28 C171A mutant was generated by site-directed mutagenesis using the following primers: 5′-AATGTTGGCAATACAGCTTGGTTTAGTGCTGTTATT-3′ and 5′-GAATAACAGCACTAAACCAAGCTGTATTGCCAACATT-3′. Flag-USP28, Myc-FBW7, HA-Ubiquitin, and Flag-BRAF 600E were purchased for Addgene. 

The following antibodies were used for immunoblotting: HA 1:1,000 (Y11, Santa-Cruz Biotech); Myc 1:1,000 (9E10, A14, Santa-Cruz Biotech); Flag 1:3,000 (Sigma); phospho-ERK 1:1,000 (T202/Tyr204, 9101, Cell Signaling); ERK1/2 1:1000 (9102, Cell Signaling); USP28 1:1,000 (HPA006778, Sigma); 1:1,000 (ab56900, Abcam); β-actin 1:10,000 (Sigma); BRAF 1:1,000 (F3, Santa-Cruz Biotech); A-RAF 1:1000 (4432, Cell Signaling); Ubiquitin 1:1,000 (P4D1, Santa-Cruz Biotech); C-RAF (9422, Cell Signaling); Bim (2819, Cell Signaling); Cleaved Caspase 3 (9661, Cell Signaling); and Cleaved PARP (9681, Cell Signaling). FBW7 (A301-720A, Bethyl) detects the first 50 amino acids in the FBW7 protein, which are not present in the Myc-FBW7 construct. PLX4032 (BRAF 600E inhibitor) was purchased from Selleckchem and dissolved in DMSO.

### Generation of CRISPR knockout cell lines

Guide RNA (gRNA) was chosen from the bioinformatically computed genome-wide resource of candidate unique gRNA targets in human exons (Mali et al., 2013) and cross-referenced with the CRISPR design program for off-target effects (Massachusetts Institute of Technology, Cambridge, MA). gRNAs with the highest guide percent on target score were chosen. PCR primers were designed incorporating a BbsI restriction enzyme cloning site, guide RNA, and sequence overlap with a module cassette containing an independent tracerRNA sequence. PCR products were digested with BbsI, purified, and cloned into px462-hSpCas9n-2A-Puro thus allowing two guide RNAs and the CAS9 enzyme to be localized on the same vector. The module cassette vector was a gift from L. Brunham and S. Sadananda (A*STAR, Singapore). 1 µg of PX462-USP28 vector and 0.1 µg of cmv-GFP were cotransfected into WM164 cells. 48 h after transfection cells were treated with puromycin for 1 wk. Cells were trypsinized and ∼1,000 cells were plated into 15-cm plates. Single green colonies were picked and expanded and Western blot was performed to determine USP28 expression. Clones displaying loss of USP28 expression were further examined. To confirm genomic alterations in the USP28 locus, PCR primers were designed surrounding the USP28 gRNA locus, and PCR amplification was performed. PCR products were subsequently transferred into TA cloning vectors, and PCR products were sequenced.

### Immunoprecipitation

Immunoprecipitation experiments were performed as in Iyengar et al. (Iyengar et al., 2015). In short, cells were lysed in ELB buffer (250 mM NaCL, 0.5% Nonidet P-40, and 50 mM Hepes, pH 7.3) and supplemented with protease inhibitors. About 500 µg of cell lysates were incubated overnight at 4°C with the indicated antibodies. The lysates were further incubated with either protein A or protein G sepharose beads (GE Healthcare) for an additional 1 h, followed by three washes with the lysis buffer. The beads were boiled in 2× SDS sample buffer and separated on SDS-PAGE gels. When appropriate, cell lysates were immunoprecipitated with anti-FLAG M2 affinity resin (Sigma) for 2 h at 4°C and then subsequent steps were followed as mentioned above.

### Cell viability and SubG1 assays

Growth curves were performed in triplicate. Viability assays with CellTiter-Glo (Promega) were performed by plating 500 cells in 96-well plates, adding drug at 24 h, and assaying 3 d after drug addition. Cell cycle and hypodiploid apoptotic cells were quantified by flow cytometry as described in (Gong et al., 1994). In brief, cells were washed with PBS, fixed in cold 70% ethanol, and then stained with propidium iodide while treating with RNase (Sigma). Quantitative analysis of sub-G1 cells was performed in a FACScalibur cytometer using the Cell Quest software (BD Biosciences).

### Tumor xenografts

Mice were maintained under the institutional guidelines set by the Vall d’Hebron University Hospital Care and Use Committee. 6-wk-old female athymic nude-*Foxn1^nu^* mice were purchased from Harlan Laboratories. Mice were housed in air-filtered laminar flow cabinets with a 12-h light cycle and food and water ad libitum. Mice were handled with aseptic procedures and allowed to acclimatize to local conditions for 1 wk before the experimental manipulations. 10^7^ A373 C.6 scrambled control or A373 C.6 USP28 knockdown cells were resuspended in PBS/Matrigel (1:1; BD Biosciences) and injected subcutaneously into the right and left flank of each mouse in 200 µl of final volume (*n* = 6 for each group; 12 tumors were analyzed for each treatment group). Treatments began when tumors reached a mean size of 250 mm^3^ and were thus considered as established growing xenografts. Mice were treated twice daily with placebo or vemurafenib (PLX4032) by oral gavage. Vemurafenib (35 mg/kg or 75mg/kg twice daily) was dissolved in 10% NMP–90% PEG, freshly formulated and administrated within 30 min. For tumor growth studies, mice were treated for 15–37 d, depending on the xenograft model and treatment regimen. Tumor xenografts were measured with calipers three times a week, and tumor volume was determined using the formula: (length × width^2^) × (π/6). At the end of the experiment the animals were anesthetized with 1.5% isofluorane-air mixture and killed by cervical dislocation. Tumors were removed 2 h after the last administration.

### IHC

IHC staining on the respective formalin-fixed, paraffin-embedded tissue sections was performed using the Leica BOND-MAX and Ventana Benchmark XT autostainers according to the conditions stated in Table 1. Tissue sections of 5 µM underwent automated deparaffinization followed by incubation with their optimized antigen retrieval solutions. Slides were then incubated with antibody as indicated in Table 1. Detection of antibody staining was performed according to manufacturer’s protocol for the detection kits used with an extension of hematoxylin counterstain extended to 10 min to ensure for a defined stain. Slides were rinsed with deionized water followed by manual mounting of coverslips. Positive and negative controls were included in each run, consisting of tissue with known expression and tissue stained without primary antibody, respectively. Quantification was assessed double blind by a trained pathologist (B. Pang) and expressed as an H-score. Melanoma samples were purchased from BioMAX-ME2082A.

**Table 1.** List of antibodies used in IHC analysis of paraffin sections with related protocols

Antibody	Manufacturer	Autostainer	Dilution	Antigen retrieval	Block (min)	Antibody incubation	Detection kit used
USP28 (HPA006778)	Sigma	BOND-MAX	1/200	pH 9, 20 min	10 min	15 min	Bond Polymer Refine Detection kit
BRAF (F3) (SC-55522)	Santa Cruz	Benchmark XT	Ready-to-use	pH 9, 64 min	None	16 min	Optiview DAB IHC Detection kit

### Lentiviral expression

To produce stable cell lines, HEK293T-FT cells were transfected with Lentiviral knockdown vectors targeting USP28 or proper control vectors along with lentiviral packaging constructs (pCMV-VSVG, pMDLg-RRE, and pRSV-REV). Viral supernatants were collected and selected melanoma cells were infected with the supernatants in the presence of Polybrene (0.01%). The cells were selected and maintained in Puromycin (1.5 µg/10 ml)-containing media.

### High throughput drug screening

To perform high content drug screening, we used a customized Selleckchem anticancer library (L-3000-01, -02, and -03). In brief, control or USP28 knockdown A373 melanoma cells were seeded into 384-well plates in volume of 50 µl. 24 h later, 0.5 µl of each compound (100 µM stock concentration) was added using liquid handler for a final concentration of 1 µM. To measure cell viability, Cell-titer Glo (Promega) was added 72 h after the treatment and luminescence signal was detected using Tecan plate reader. The assay was performed three times in triplicate.

### Statistical analyses

All statistics were calculated using GraphPad Prism or Microsoft Excel. The tests used include two-tailed *t* test, standard deviation, standard error, Spearman’s analysis and nonlinear regression, extra sum-of-squares test (Shu et al., 2016), and χ^2^ test where relevant are indicated in the respective figure legends. For IHC, expression was quantified by a pathologist, blinded to the identity of the samples, using a four-value intensity score (0, 1^+^, 2^+^, 3^+^; H-score) and the percentage of the reactivity extent. A final consensual score was obtained by multiplying both intensity and extension values (range, 0–300). P-values ≤ 0.05 were considered statistically significant. All p-values are depicted in the figures or in the figure legends. All immunoblotting experiments were performed in triplicate unless otherwise indicated.

### Online supplemental material

Fig. S1 shows USP28 as a novel regulator of MAPK signaling. Fig. S2 shows that USP28/FBW7 forms a complex with RAF family members. Fig. S3 shows vemurafenib alters FBW7 ubiquitination leading to decreased BRAF stability. Fig. S4 shows the characteristics of the study cohorts used for Kaplan Meier analysis. Fig. S5 shows the sensitivity of parental cell lines and USP28 knockdown cells to vemurafenib and the loss of USP28 decreases time to progression in melanoma patients treated with MAPK pathway inhibitors. Table S1 shows the values of deubiquitinating enzyme screen analyzing altered pERK/ERK ratios. Table S2 shows USP28 copy number variation and NRAS, BRAF, NF1, and FBW7 mutation status in 118 melanoma patients. Table S3 shows the chemical compound screen in A373 cells and A373 USP28 knockdown cells.
